# Gauging the Positive Predictive Value of Exercise Tolerance Test Using Angiographic Evaluation: A Cross-Sectional Analysis From a Developing Country

**DOI:** 10.7759/cureus.12173

**Published:** 2020-12-19

**Authors:** Ismail Khan, Maria Hasan, Javeria Hasan, Ali Imran Dhillon, Moosa Khan, Mehwish Kaneez

**Affiliations:** 1 Cardiology, Pakistan Institute of Medical Sciences, Islamabad, PAK; 2 Cardiology, Rawalpindi Medical University, Rawalpindi, PAK; 3 Pharmacology, Shaheed Zulfiqar Ali Bhutto Medical University, Islamabad, PAK; 4 Internal Medicine, Rawalpindi Medical University, Rawalpindi, PAK

**Keywords:** positive predictive value, exercise tolerance test, angiographic evaluation

## Abstract

Background

Exercise tolerance test (ETT) and angiographic evaluation are important tools to evaluate patients presenting with ischemic cardiac pathologies. Angiographic evaluation is regarded as the gold standard diagnostic modality to diagnose coronary artery disease (CAD). Our study aims to evaluate the positive predictive value (PPV) of ETT to diagnose CAD using coronary angiography.

Methods

We conducted a cross-sectional study that analyzed 94 patients with a positive ETT test after the application of strict inclusion and exclusion criteria. All 94 patients were referred for angiography after a positive ETT test. Data collection was performed using a structured proforma, and analysis was carried out on Statistical Package for Social Sciences (SPSS) version 23 (IBM Corp., Armonk, NY). PPV for various demographic characteristics was calculated.

Results

Out of 94 patients, 76 were males and 18 were females with a mean age of 52.28 ± 7.55 years. A total of 35.1% of the patients had type-2 diabetes, and 31.9% were hypertensive. On coronary angiography, only 25 patients had normal findings, and 69 patients had a significant occlusion in at least one of the major coronary arteries. The overall PPV of the ETT against angiographic evaluation was 73.40%. The PPV for females, hypertensives, non-smokers, and non-diabetics was lower than the PPV of males, smokers, non-hypertensives, and diabetics.

Conclusion

Angiographic evaluation of patients with positive ETT findings has a high likelihood of false positivity especially among females, non-smokers, hypertensives, and non-diabetics. The results of ETT must be interpreted with caution in these subsets of the population. Invasive radiological modalities can be used for diagnosis; however, such modalities do not elucidate the functioning of myocardium under stress.

## Introduction

Coronary artery disease (CAD) is attributed as a common cause of mortality and morbidity, especially in middle-aged adults [1]. Exercise tolerance test (ETT) is a frequently used investigation that utilizes a series of exercise tests and electrocardiography to evaluate ischemic cardiac pathologies [2]. ETT is the first-line investigation for CAD, which is used to decide which patient requires a referral to a cardiologist for further workup [3]. It has high sensitivity; however, it is less specific because various other cardiovascular and endocrinological diseases may influence its outcome [4,5]. Nonetheless, its role as a preliminary and non-invasive investigation in the early detection of ischemic cardiac pathologies is well established [5]. To perform an accurate ETT, multiple protocols have been suggested, of which the most commonly used is the Bruce protocol [6,7]. The progressive increase in exercise at different stages helps in recognizing the ischemic changes in the myocardium and contributes to the high sensitivity of Bruce protocol [6,8]. ETT is performed to appraise the health, diagnose heart or lung pathologies, induce angina, and gauge the stability of the cardiopulmonary system [8]. The test is contraindicated in the ongoing unstable angina, myocardial infarction, ventricular arrhythmias, hemodynamic instability, aortic aneurysm, aortic dissection, and pulmonary embolism [9].

Even though, in literature, the validity and reliability of a diagnostic test are represented by its sensitivity and specificity, in clinical practice, a physician is more interested in finding the disease in whom he or she suspects the disease which is represented by the positive predictive value (PPV) of a diagnostic test [10]. Thus, a physician is much more concerned with the possibility of the people actually having the disease (true-positives) of those who tested positive.

A high PPV of a diagnostic test helps in avoiding unnecessary admissions, financial burden, anxiety, and stress. However, most of the studies previously done in cardiology show workup, setup, or referral inclination because they are done in specialty practice in a different setting. Pertinently, ETT is improperly utilized due to a lack of awareness and observer bias resulting in inappropriate diagnosis and treatment [6,10]. Very few studies have been carried out to evaluate the PPV of ETT in developing countries. Our study will add up to the growing literature of exercise testing and factors influencing its results in a developing country.

## Materials and methods

A prospective cross-sectional study was conducted in the Cardiology Department of Pakistan Institute of Medical Sciences (PIMS), Islamabad, Pakistan. A total of 214 patients who had positive ETT for inducible angina during the study period of January 2018 till May 2019 were enrolled in the study. Patients with previously diagnosed ischemic heart disease, those with previous abnormal angiography, and the ones having contraindications to angiography were excluded. Furthermore, patients with a history of percutaneous coronary intervention (PCI), coronary artery bypass graft (CABG) surgery, and valvular replacement were also excluded. Pertinently, patients with known valvular disorders, Wolf-Parkinson's-White (WPW) syndrome, or who were suffering from any obstructive or restrictive lung pathology were also subjected to exclusion. After the application of these strict exclusion criteria, a total of only 94 patients were a part of the final analysis.

All selected 94 patients fulfilling the inclusion and exclusion criteria underwent angiography, and their results were entered in a predefined proforma. Consent was obtained from all study participants, and confidentiality was maintained. The sample size was obtained using an openEpi (developed by AG Dean, KM Sullivan, and MM Soe) sample size calculator. Ethical considerations were certified by the Ethical Review Committee of PIMS. The baseline demographic characteristics of the study participants were tabulated among patients with true-positive and false-positive test results. PPVs for each demographic variable were calculated. The chi-square test was used to assess any significant association between categorical study variables. A p value of less than 0.05 was considered statistically significant. Data were analyzed using Statistical Package for Social Sciences (SPSS) version 23 (IBM Corp., Armonk, NY).

## Results

In the present study analyzing 94 patients, 76 were males and 18 were females with ages raging between 27 and 68 years. There was a diffuse distribution of risk factors including diabetes, hypertension, and smoking. The baseline demographics of the study participants are shown in Table 1.

**Table 1:** Background characteristics of the study participants. SVCAD: Single-vessel coronary artery disease; DVCAD: double-vessel coronary artery disease; TVCAD: triple-vessel coronary artery disease; CABG: coronary artery bypass grafting.

Parameters	Results
Total Patients	94
Age Range	27–68 years
Mean Age	52.28 ± 7.55 years
Gender Distribution	Males: 76 (80.85%)
	Females: 18 (19.15%)
Diabetics	33 (35.1%)
Hypertensive	30 (31.9%)
Smokers	9 (9.57%)
Angiography Results	Normal: 25 (26.6%)
	SVCAD: 34 (36.2%)
	DVCAD: 13 (13.8%)
	TVCAD: 22 (23.4%)
Treatment Offered	Angioplasty: 43 (45.7%)
	CABG: 15 (16%)
	Medical Treatment: 12 (12.8%)
	Reassurance: 24 (25.5%)

The mean age of patients with positive ETT and significant vessel occlusion on angiographic evaluation (true-positives) was 52.49 ± 6.75, whereas those with positive ETT and normal angiography (false-positives) were 51.68 ± 9.56. A comparison of true-positives and false-positives concerning baseline characteristics are delineated in Table 2.

**Table 2:** Comparison between true-positives and false-positives of ETT. ETT: Exercise tolerance test. *Chi-square test.

Parameters		True-Positive	False-Positive	P Value*
Diabetes	Diabetics	26 (37.7%)	7 (28%)	0.39
	Non-diabetics	43 (62.3%)	18 (72%)	
Smoking Status	Smoker	9 (13.04%)	0 (0%)	0.05
	Non-smoker	60 (86.96%)	25 (100%)	
Gender	Males	60 (87%)	16 (64%)	0.01
	Females	9 (13%)	9 (36%)	
Hypertension	Hypertensive	17 (24.6%)	13 (52%)	0.02
	Non-hypertensive	52 (75.4%)	12 (48%)	
Stage of ETT	Stage 1	23 (33.3%)	4 (16%)	0.17
	Stage 2	25 (36.2%)	9 (36%)	
	Stage 3	21 (30.4%)	12 (48%)	

The overall PPV of ETT using angiographic evaluation was 73.40%. The PPVs of various other study parameters are elucidated in Table 3.

**Table 3:** A delineation of positive predictive values of various parameters. ETT: Exercise tolerance test.

Parameters		Positive predictive values (PPV)
Diabetes	Diabetics	78.8%
	Non-diabetics	70.5%
Smoking Status	Smoker	100%
	Non-smoker	70.6%
Gender	Males	78.9%
	Females	50%
Hypertension	Hypertensive	56.6%
	Non-hypertensive	81.2%
Stage of ETT	Stage 1	85.2%
	Stage 2	73.5%
	Stage 3	63.6%

## Discussion

ETT is a validated and affordable diagnostic test employed for the evaluation of patients with CAD for years. We evaluated the angiography results of 94 consecutive patients who were enrolled after they were labeled positive for inducible angina on ETT via the Bruce protocol. The literature regards ETT as an initial measure to assess and rule out myocardial ischemia [2,3,11]. The overall sensitivity of this inexpensive test is reported to be as high as 98%; however, its specificity is quite low [5]. Studies have reported that low specificity can be attributed to a myriad of contributing parameters including metabolic diseases, structural heart diseases, conduction disturbances, and digitalis therapy [3,5]. In developing countries, further evaluation in patients with coronary artery disease hinges upon the results of ETT, a relatively inexpensive and easy to interpret test [12,13]. Thus, patients with CAD cannot be ruled out without considering the probability and predictive value of the diagnostic test.

In our study, 73.4% of patients with positive ETT had abnormal findings on angiography. This finding is consistent with other studies that reported a PPV of ETT between 75% and 85% [4,5]. In another study, the PPV of ETT was estimated to be 78% with ST-segment depression of 1.0 to 1.9 millimeters (mm) and 97% with ST-segment depression of 2 mm or greater [6]. Similarly, the PPV of ETT improves when multiple variables are considered rather than a single variable [4].

Approximately 21% of the male patients and 50% of the female patients had a normal coronary angiogram and were labeled as false-positive ETT results. Thus, the PPV of males (78.9%) was found to be significantly greater than females (50%) with a significant p value of 0.01. A study done in Bangladesh concluded that the ETT was more sensitive for males, while the females were prone to have false-positive results in this regard [12]. Furthermore, another study reports that females have a greater risk of false-positive ETT, which has led to the use of more invasive radiological modalities (magnetic resonance imaging or radioactive scans) for initial evaluation of CAD [14,15]. Pertinently, women have a higher predisposition toward false-positive results, and they should be investigated and managed with caution, and further evaluation should be considered [15]. The low PPV dramatically affects the efficacy and accuracy of a test. Hence, a greater number of false-positives cause an increased patient load, psychological stress, and an overall elevated economic burden on the healthcare system especially in developing countries [10].

Previous literature reported PPV among diabetics to be hovering at 77% that matches the results of our study [16,17]. Our study also advocates that chronic smokers have higher PPV than non-smokers. Identification of diabetes and chronic tobacco smoking as established risk factors for the development of complex CAD may explain the higher PPV in these subsets of patients [16]. Furthermore, the number of false-positives in hypertensives was more than normotensives. Another study reported the PPV of hypertensives to be lower (50%) than the normotensives (88.2%) [13]. The probable reason for low PPV in hypertensives is ST-segment depression due to impaired subendocardial perfusion leading to an elevated number of false-positives [13].

Thus, the restricted validity of the ETT for detection of inducible angina has led to an increase in the use of invasive technologies such as myocardial perfusion scan (MPS) or cardiac magnetic resonance imaging (CMR) [18]. Nevertheless, ETT is still an important tool that aids to understand the functional capacity of the heart during exercise and stress with normal resting ECG [19].

## Conclusions

ETT is a useful yet unreliable diagnostic test for the early detection of CAD. Coronary angiography showed that positive exercise tolerance of ETT had a high likelihood of false positivity, especially in females, non-smokers, and hypertensive patients. The PPV of ETT for smokers and diabetic patients was higher than the PPV for non-smokers and non-diabetic patients. Diagnostic modalities like MPS and CMR can also be utilized to better evaluate CAD.
